# Focusing on perihematomal hypoperfusion following intracerebral hemorrhage: from oxidative stress to prospective therapeutic approaches

**DOI:** 10.3389/fphys.2025.1646959

**Published:** 2025-08-05

**Authors:** Huang Zhipeng, Liu Shan, Zhang Dechou, Li Shuangyang, Bai Xue

**Affiliations:** ^1^ College of Integration of Traditional Chinese and Western Medicine, Southwest Medical University, Luzhou, China; ^2^ Institute of Integrated Chinese and Western Medicine, Southwest Medical University, Luzhou, China

**Keywords:** intracerebal hemorrhage, perihematomal hypoperfusion, oxidative stress, therapeutic approaches, endothelial dysfuction

## Introduction

Intracerebral haemorrhage (ICH), while only constituting 10%–15% of strokes, is associated with significantly elevated disability and mortality rates, with survivors often experiencing profound and enduring neurological impairment. It is essential to elucidate the pathogenic mechanisms of subsequent injury following ICH and preserve the function of the remaining brain tissue. Oxidative stress is a key mechanism of neurological damage after intracerebral hemorrhage. This letter focuses on the pathophysiological basis and potential antioxidant strategies to ameliorate hypoperfusion injury following ICH, hoping to illustrate novel perspectives on the therapy of this condition.

Hemorrhage and ischaemia appear to be contradictory, but just as hemorrhagic transformation can occur in cerebral infarction, hypoperfusion has a negative impact on the responsiveness and self-regulation of cerebral vessels, which is directly related to the poor prognosis of patients with cerebral haemorrhage (Zhang et al., 2022). Perihematomal tissues in ICH have persistent hypoperfusion within 1 week of onset and even in the chronic stage, which can be observed in neuroimaging as decreased diffusion-perfusion, decreased regional cerebral blood flow (rCBF) and regional cerebral blood volume (rCBV), and delayed mean transit time (MTT) and time-to-peak (TTP) adjacent to hematoma (Kidwell et al., 2001; Zhou et al., 2010). The self-regulation of cerebral blood flow (CBF) ensures that blood flow to the brain remains stable even when systemic blood pressure changes, preventing ischemic damage when hemodynamics are unstable. However, the hematoma and its consequent cerebral edema can alter oxygen and nutrition flow to afflicted brain regions, complicating prediction models due to the intricate connections between risk factors and clinical consequences.

**FIGURE 1:**
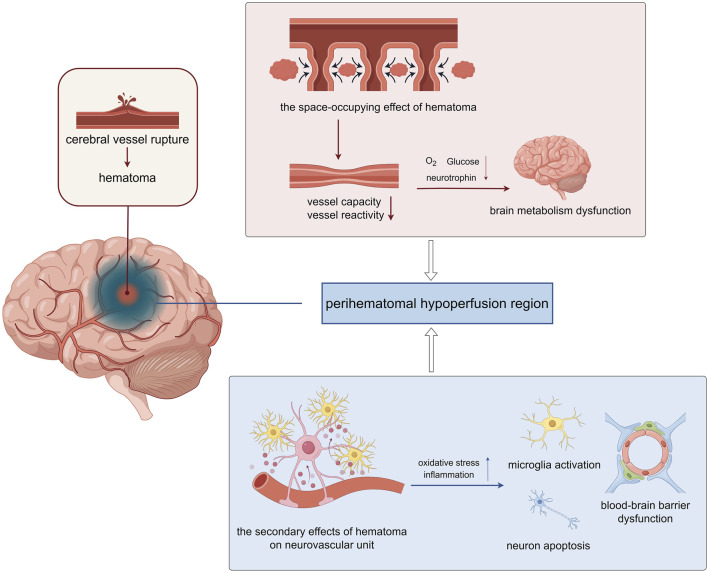
The perihematomal hypoperfusion injury following intracerebral hemorrhage (ICH) involves two primary pathological mechanisms. Mechanical compression from the expanding hematoma reduces both the vascular bed volume and vasoreactivity in surrounding tissues, leading to compromised cerebral perfusion. This hemodynamic alteration results in inadequate delivery of oxygen and metabolic substrates to the perihematomal region, ultimately causing localized cerebral metabolic dysfunction. The hematoma exerts significant cytotoxic effects through multiple pathways. The extravasated blood components trigger a cascade of pathological responses, including oxidative stress and neuroinflammatory reactions. These processes promote microglial activation, induce neuronal apoptosis, and disrupt the structural integrity of the blood-brain barrier (BBB) through degradation of tight junction proteins. Consequently, the compromised BBB function exacerbates neuronal injury and contributes to the progression of secondary brain damage (The figure was illustrated by https://www.figdraw.com).

CBF is regulated by vascular baroreflex, renin-angiotensin system and endothelial nitric oxide release. Small intracranial vessels change vascular resistance through contraction and relaxation. Through changes in physiological needs and compensatory pathological changes, CBF balance is dynamically maintained to maintain oxygen and energy supply in the brain (Xiao et al., 2017). After cerebral vessel rupture, on the one hand, the hematoma formed has a significant space-occupying effect, and could directly compress surrounding brain tissue. The mechanical space-occupying compression of hematoma causes a rapid increase in intracranial pressure, which is first augmented by reflex hypertension and CBF pressure. However, the hematoma and perihematoma edema directly mechanically compress the vessels of the surrounding tissues, obstruct venous return and cause a decrease in the volume of the surrounding vascular bed. These alternations lead to the reflex dysfunction of the vessels and increased intracranial pressure compresses the lesions around the hematoma, resulting in microcirculation disorders. And the toxic reaction caused by hematoma components and their decomposition metabolites can result in secondary damage, such as neuroinflammation, brain edema and worsening high intracranial pressure, and interfere with neurovascular coupling, tissue hypoxia and potentially irreversible biochemical damage (Figure 1).

In the formation and progression of ICH hypoperfusion, the space-occupying effect of the hematoma and its secondary effects can directly or indirectly damage various cellular components in the central nervous system, including inducing endothelial cell dysfunction, neurotoxicity leading to neuronal apoptosis, and abnormal polarization of microglia aggravating neuroinflammation. Cerebral endothelial cell (CEC) is the core of blood perfusion and the basis of blood-brain barrier (BBB. It helps to maintain cerebral homeostasis. CEC can respond quickly and compensatorily regulate CBF in the early phases of cerebral perfusion alterations by sensing mechanical compression, blood flow shear stress, and other mechanisms. Hypoperfusion causes a decrease in oxygen and glucose supply, blocks the synthesis of adenosine triphosphate (ATP), and impairs endothelial mitochondrial function. And the failure of the cell membrane ion pumps cause cerebral microvascular endothelial dysfunction, inducing neurovascular uncoupling and diminished oxygenation capacity. Meanwhile, as the main component of the BBB, pathological reactions, including neuroinflammation and oxidative stress, can damage the selective transport function and tight junctions of CECs, allowing neurotoxic substances such as peripheral cytokines to enter the central nervous system, further aggravating neurological damage. Additionally, the above pathological and physiological links do not appear alone or step by step, but interact with each other to form a vicious circle. Therefore, focusing on improving the CEC function is of great value in improving perihematomal hypoperfusion injury.

The secondary damage of ICH is the primary connection to the subsequent impact of neurological impairment. Endothelial dysfunction following ICH results in inadequate bioavailability of nitric oxide (NO), which is deactivated by its interaction with superoxide anions, producing superoxynitrite. The disproportion between the production and elimination of reactive oxygen free radicals leads to oxidative stress. An abundance of free radicals exacerbates CEC dysfunction by impairing DNA, disrupting signal transduction, inducing cell apoptosis and autophagy, and influencing gene expression regulation. This culminates in CBF imbalance and BBB damage, while also facilitating the activation of immune cells within the central nervous system, thereby creating an inflammatory milieu that further deteriorates endothelial function and exacerbates BBB compromise and abnormal brain perfusion. Recent investigations also indicate that endothelin-1, soluble nicotinamide adenine dinucleotide 2-derived peptide (sNOX2-dp), and asymmetric dimethylarginine may contribute to the development of hypoperfused tissue surrounding the hematoma (De Michele et al., 2024).

Tetrahydrobiopterin (BH4) is an important cofactor of nitric oxide synthase (NOS) and aromatic amino acid hydroxylase, and is involved in the production of endogenous vasodilator NO. It has physiological functions such as inhibiting platelet aggregation and adhesion, inhibiting leukocyte adhesion and vascular inflammation, inhibiting smooth muscle cell proliferation, and anti-atherosclerosis, and plays an important role in maintaining the normal function of the cardiovascular system (Edwards and Farquhar, 2015; Zhao et al., 2023; Ohashi et al., 2016). NOS in endothelial cells is divided into endothelial and inducible types, which widely involved in inflammatory response, angiogenesis, immune response, atherosclerosis and vascular tension regulation (Liu et al., 2021). Endothelial dysfunction mediated by eNOS uncoupling is believed to be caused by increased superoxide anion synthesis (through NADPH oxidase-dependent mechanisms), increased ONOO- formation, and decreased BH4 levels, which in turn reduces NO synthesis (Najjar et al., 2013). Functionally, NOS, which is dependent on BH4 regulation, is crucial in cardiovascular diseases. After coupling with NOS, BH4 can oxidize L-arginine to L-guanidine and produce NO. The generated NO can regulate vasoconstriction and relaxation. However, because NOS lacks BH4, it is not inactive, but becomes “uncoupled”. The electrons from NADPH are transferred through the flavin in the reductase domain and continue to form blood oxygen intermediates at the active site. If there is no BH4, the reduction of blood oxygen intermediates is not coupled with the oxidation of L-arginine, so superoxide anions are produced instead of NO (Channon, 2012). In summary, BH4 deficiency can lead to endothelial dysfunction. In the central nervous system, BH4 is a cofactor required for the enzymatic conversion of monoamine precursors tryptophan and tyrosine by phenylalanine hydroxylase (PAH). Tryptophan hydroxylase and tyrosine hydroxylase are essential for the biosynthesis of neurotransmitters serotonin, dopamine, and norepinephrine. BH4 deficiency affects neuronal function, manifesting as movement disorders, emotional regulation disorders, cognitive disorders, sleep disorders, and other symptoms (Leblhuber et al., 2021; Sur et al., 2023). BH4 is a very important antioxidant that protects cells from oxidative damage by reacting with free radicals and peroxides (Cerrah et al., 2023). In addition, some studies have also found that BH4 can exert its antioxidant effect by regulating the synthesis of coenzyme Q10, thereby inhibiting lipid peroxidation and iron allergy (Cheng et al., 2023). In summary, BH4 plays an important antagonistic role in oxidative stress, participating in the antioxidant defense response of cells through multiple mechanisms, thereby increasing the bio-availability of NO, reducing the damage of oxidative stress to neurons, regulating local tissue blood perfusion and avoiding damage to the nervous system. Therefore, promoting the production level of BH4 in clinical practice can help prevent or treat neurological dysfunction and low blood perfusion caused by oxidative stress.

Supplementation of BH4 seems to be one of the ideal ways to ameliorate oxidative stress injury in perihematomal tissue. However, due to the metabolic effects of the kidney, there is still much controversy about the ability of BH4 to enter the central nervous system and its oral bioavailability after peripheral administration (Ohashi et al., 2012). At the same time, the cost of BH4 synthesis is relatively high, which makes the clinical value of supplementing BH4 from the periphery limited. However, from the perspective of the biochemical processes involved in BH4 (Ohashi et al., 2011), increasing BH4 synthesis or limiting its oxidative degradation may be a more ideal and easily achievable strategy. Previous studies have pointed out that 5-methyltetrahydrofolate, the active metabolite of folic acid, can reduce the oxidation of BH4 by increasing DHFR activity and increase the recycling of BH2 to BH4 (Chalupsky et al., 2015; Hyndman et al., 2002). At the same time, recent clinical studies have proved that phototherapy can significantly increase the serum BH4 level and regulate neuroinflammatory response in patients with post-stroke depression (Liu et al., 2024; Xie et al., 2025). In addition, some compounds derived from natural herbs, such as Berberine, have been reported to increase BH4 content and thus improve vascular endothelial cell damage (Yin et al., 2019). These evidences provide insights for antioxidant adjuvant therapy in ICH.

Superoxide dismutase (SOD) is a type of antioxidant metalloenzyme widely distributed in tissues and cells. It catalyzes the dismutation of superoxide anion free radicals to generate O_2_ and H_2_O_2_. And plays an important regulatory role in oxidation and antioxidant systems, as well as removing free radicals (Liu et al., 2022). Malondialdehyde (MDA) is one of many lipid oxidants. It is a metabolite produced by free radicals attacking and oxidizing polyunsaturated fatty acids in cell membranes including neurons. It represents the degree of lipid peroxidation caused by free radicals attacking nerve cells (Haro Giron et al., 2023). Brain edema and peripheral ischemia after intracerebral hemorrhage can cause an increase in the level of free radicals around the hematoma, promote the activation of matrix metalloproteinases (MMPs), pathologically increase the permeability of the BBB, and increase the risk of intracerebral hemorrhage and brain edema (Xu et al., 2021). At the same time as neurological deficits occur in intracerebral hemorrhage, brain water content increases, brain tissue MDA content increases significantly, and SOD activity decreases significantly. During intracerebral hemorrhage, lipid peroxidation reactions are enhanced and the activity of anti-free radical enzymes decreases, further confirming that free radicals are related to brain tissue damage after intracerebral hemorrhage. The SOD content indirectly reflects the ability to neutralize reactive oxygen species (ROS), while the MDA content reflects the severity of the exposure to free radicals. In clinical treatment, experimental studies have shown that hyperbaric oxygen can reduce the production of free radicals after intracerebral hemorrhage by regulating SOD levels, inhibit the excessive production of NO after cerebral hemorrhage, and enhance ATPase activity, thereby further reducing the occurrence of cerebral edema (Wada et al., 2000; Narkowicz et al., 1993).

However, due to the invasiveness and poor repeatability of detection like computed tomography perfusion imaging, non-invasive examinations such as transcranial Doppler (TCD) and arterial spin labeling (ASL) can resolve the current controversy over the formation and duration of the perihematomal hypoperfusion. ASL uses magnetized labeled arterial blood water protons as endogenous tracers, which can quickly and accurately detect local or regional cerebral blood flow without the use of contrast agents. This is especially suitable for evaluating CBF in the initial stage of onset or the stage of disease progression, and various studies have used ASL to focus on the CBF in ICH (Lee et al., 2021; Klahr et al., 2019; Klahr et al., 2018). TCD deduces alterations in focal CBF from variations in CBF velocity, but with marginally lower accuracy than ASL. Nonetheless, owing to the ease of the detection apparatus, it can be utilized for real-time bedside monitoring or prolonged assessment, and it has been extensively employed in both clinical and research (Chen et al., 2018; Fulesdi et al., 2014; Ke et al., 2012). This will enhance comprehension of the pathophysiological mechanisms underlying perihematomal hypoperfusion and ascertain the optimal intervention time. It is especially eessential for the development of sequential and complementary therapies in ICH. This includes the intensity of blood pressure control in the ultra-early and subacute phases and the duration of maintenance of different blood pressure levels. Whether it is reasonable to maintain a rather high blood pressure during the hypoperfusion period to ensure enough cerebral blood perfusion is worth considering (Carteron et al., 2017). Meawhile, investigating strategies to enhance the oxygenation capacity in patients with ICH, whether through staged hyperbaric oxygen therapy or the development of drugs to improve oxygenation capacity, is a promising direction. While perihematomal hypoperfusion and cerebral ischaemia share some common mechanisms, there are significant disparities in the pathogenic environment of the hypoperfusion region due to their distinct pathogens. Future study is necessary to evaluate the efficacy of vascular active medicines for neurovascular protection in ICH. The development of small molecules or carrier medications that particularly safeguard the function of vascular endothelial cells in hypoperfused regions and mitigate BBB damage surrounding the haematoma is highly valuable for preserving blood perfusion and preventing subsequent damage.
